# FungalTraits vs. FUNGuild: Comparison of Ecological Functional Assignments of Leaf- and Needle-Associated Fungi Across 12 Temperate Tree Species

**DOI:** 10.1007/s00248-022-01973-2

**Published:** 2022-02-05

**Authors:** Benjawan Tanunchai, Li Ji, Simon Andreas Schroeter, Sara Fareed Mohamed Wahdan, Shakhawat Hossen, Yoseph Delelegn, François Buscot, Ann-Sophie Lehnert, Eliane Gomes Alves, Ines Hilke, Gerd Gleixner, Ernst-Detlef Schulze, Matthias Noll, Witoon Purahong

**Affiliations:** 1 Department of Soil Ecology, UFZ-Helmholtz Centre for Environmental Research, Theodor-Lieser-Str. 4, 06120 Halle (Saale), Germany; 2 Bayreuth Center of Ecology and Environmental Research (BayCEER), University of Bayreuth, Bayreuth, Germany; 3 Key Laboratory of Sustainable Forest Ecosystem Management-Ministry of Education, School of Forestry, Northeast Forestry University, 150040 Harbin, People’s Republic of China; 4 Max Planck Institute for Biogeochemistry, Biogeochemical Processes Department, Hans-Knöll-Str. 10, 07745 Jena, Germany; 5 Botany Department, Faculty of Science, Suez Canal University, Ismailia, 41522 Egypt; 6 Institute of Bioanalysis, Coburg University of Applied Sciences and Arts, Coburg, Germany; 7 German Centre for Integrative Biodiversity Research (iDiv) Halle-Jena-Leipzig, Deutscher Platz 5e, 04103 Leipzig, Germany

**Keywords:** Amplicon sequence variants, Endophytes, Functional assignment, Fungal amplicon sequencing, ITS, Lichenized fungi, Plant pathogens, Saprotrophs

## Abstract

Recently, a new annotation tool “FungalTraits” was created based on the previous FUNGuild and Fun^Fun^ databases, which has attracted high attention in the scientific community. These databases were widely used to gain more information from fungal sequencing datasets by assigning fungal functional traits. More than 1500 publications so far employed FUNGuild and the aim of this study is to compare this successful database with the recent FungalTraits database. Quality and quantity of the assignment by FUNGuild and FungalTraits to a fungal internal transcribed spacer (ITS)–based amplicon sequencing dataset on amplicon sequence variants (ASVs) were addressed. Sequencing dataset was derived from leaves and needles of 12 temperate broadleaved and coniferous tree species. We found that FungalTraits assigned more functional traits than FUNGuild, and especially the coverage of saprotrophs, plant pathogens, and endophytes was higher while lichenized fungi revealed similar findings. Moreover, ASVs derived from leaves and needles of each tree species were better assigned to all available fungal traits as well as to saprotrophs by FungalTraits compared to FUNGuild in particular for broadleaved tree species. Assigned ASV richness as well as fungal functional community composition was higher and more diverse after analyses with FungalTraits compared to FUNGuild. Moreover, datasets of both databases showed similar effect of environmental factors for saprotrophs but for endophytes, unidentical patterns of significant corresponding factors were obtained. As a conclusion, FungalTraits is superior to FUNGuild in assigning a higher quantity and quality of ASVs as well as a higher frequency of significant correlations with environmental factors.

**Supplementary Information:**

The online version contains supplementary material available at 10.1007/s00248-022-01973-2.

## Introduction

Fungi play a pivotal role in terrestrial ecosystems and exert important ecological functions including decomposition, transformation, and effective utilization of organic substrates, facilitating the cycling processes [1]. The understanding of fungal community diversity associated with leaf decomposition provides new insights into changes in biodiversity and forest ecosystem functions under climate change scenarios [2, 3]. Leaf properties (especially pH, availability of N and C nutrients) vary greatly among different host tree species and tree types and are known to shape the microbial community composition and microbial functional groups [4–6]. However, little attention has been paid to the fungal community functions related to leaves and needles of temperate tree species [7] and their relation to the host tree species. A functional guild summarizes a functional group composed of different phylogenetic taxa, which employs similar utilization of the same substrate type. Nevertheless, not all fungal groups perform consistent ecological functions, potentially resulting in guild bias in the relationships between the relative abundance of fungal communities and ecological functions. Some fungal groups even have overlapping niches under certain conditions [8–10]. Less is known until recently on how different fungal guilds interplay in facilitating and competitive modes in different host tree species. The interaction between fungal guilds can impact the decomposition rate of organic matter through the priming effect (additional carbon input) or the Gadgil effect (competition between saprotrophs and ectomycorrhizas for limited organic resources) [11–13]. Saprotrophs and plant pathogens are the two main guilds inhabiting the leaf litter. Saprotrophs exert a variety of functions in forest debris (e.g., dead wood and leaf litter), soil carbon, and nutrient cycling [14], while plant pathogens usually exhibit saprophytic activities after leaf senescence (in the early stage of decomposition) [15]. These characteristics can lead to differences of the functional richness between the saprotrophs and plant pathogens in different habitats. Therefore, identifying and evaluating how fungal guilds and their richness in the respective functional diversity response to the variation in host tree species and diversity is a crucial issue for microbial ecology and biodiversity. The functional assignment to phylogenetic datasets is an important step to assess fungal community functions and guild differentiation.

In recent years, several relatively efficient and accurate databases or molecular tools that depict and identify fungal functions have been established, for example LIAS [16, 17], DEEMY [18], Fun^Fun^ [19], Notes on genera: Ascomycota [20], FUNGuild [21], and FacesOfFungi [22]. LIAS focuses on lichens, lichenicolous fungi, and non-lichenized Ascomycetes, whereas DEEMY focuses on ectomycorrhizal fungi [16, 18]. FacesOfFungi is a broad database and includes three main fungal groups, Ascomycota, Basidiomycota, basal fungi as well as fungus-like organisms [22]. Notes on genera: Ascomycota has been built up from FacesOfFungi by focusing on habitats, substrates, gross biotic interactions, and trophic modes of Ascomycetes [20]. FUNGuild is a database for the comparison of fungal functions and can link fungal gene sequencing information with the ecological functions of fungi, as well as identify the nutrient types used by fungi at the genus level and conduct the specific functional classifications [21]. Fun^Fun^ database has been developed from FUNGuild by addition of data on various number of traits at the genus and species levels (including cellular, ecological, and biochemical traits) [19]. The FUNGuild annotation tool proposed by Nguyen et al. (2016) for analyzing the functional guilds of fungal communities [21] has received more attention and has been applied to perform the fungal ecological functions in terrestrial and aquatic ecosystems [23–28]. The script is written in Python and licensed under GNU General Public License. FUNGuild’s script works by matching terms in the taxonomy column of the OTU table to those in the database in the GitHub repository [21]. Even though FUNGuild has been used to analyze the functions of fungi to a certain extent, the functions of 59% of soil fungi and 20% of saprophytic organisms have not yet been resolved [21]. This tool has certain limitations as it is based on existing literature and data. Therefore, the fungal taxon and functional group datasets still need to be updated with higher resolution.

Based on the previous fungal functional annotation tools, FUNGuild [21] and Fun^Fun^ [19], the recent work by Põlme et al. developed the FungalTraits tool and reannotated 10,210 genera of fungi and 151 genera of Stramenopila associated with 17 characteristic lifestyles [29]. They manually classified and assigned the 697,413 fungal ITS sequences and obtained the 92,623 fungal characteristics and host information (at 1% dissimilarity threshold). Compared with FUNGuild, FungalTraits clearly provides the most commonly occurring lifestyle as primary lifestyle and additional relevant lifestyle as secondary lifestyles. Furthermore, FungalTraits introduced the “aquatic_habitat” feature, which allows fungi to be classified as marine, freshwater, more extensive aquatic, or partial aquatic organisms, because many previous aquatic species were usually annotated to root or soil environments. They unravelled that it may be necessary to parse accidental spores of terrestrial fungi from functional groups that naturally grow in water or similar substrates [29]. In addition, FungalTraits has also expanded the “growth_form” field, with 15 characteristic states covering amoeba, filamentous, mycelium, and various single-cell forms related to fungi and Stramepiles. For ectomycorrhizal fungi, the evolutionary characters “ectomycorrhiza_lineage” and “ectomycorrhiza_exploration_type” are introduced. In addition, they collected specific information about primary and secondary symbiotic photosynthetic organisms in the literature to annotated information about lichen traits [29].

Until now, more than 1500 publications (last accessed 16.12.2021) have employed FUNGuild to annotate sequencing datasets to ecological functions. Due to the large number of previous studies using FUNGuild, it is necessary to compare the performance and the ecological interpretation provided by the two annotation tools (FUNGuild vs. FungalTraits). Our study aimed to compare state-of-the-art methods (FungalTraits vs. FUNGuild) for leaf- and needle-associated fungal functional diversity analyses. We investigated the leaf- and needle-associated fungi of 12 temperate tree species in Central Europe forests and validated the consistency of using the two functional annotation tools. We hypothesize that (1) for overall and for all main fungal guilds, FungalTraits outperforms FUNGuild as it contains a higher number of fungal genera in its database; (2) both functional annotation tools provide consistent results for interpreting the richness and community composition of the leaf- and needle-associated fungi across 12 temperate tree species.

## Materials and Methods

### Study Site and Sampling

This study was conducted in the Hainich-Dün region of Thuringia, Germany (51°12' N 10°18'E). Elevations range from 100 to 494 m above sea level, the mean annual precipitations from 600 to 800 mm, and the mean annual temperatures from 6 to 7.5 °C (average temperatures in January = 0.65 °C and July = 17.17 °C). The parent material is Triassic limestone, which is covered by a Pleistocene loess layer of variable thickness (ca. 10–50 cm) at most sites. The litter layer consists mainly of past years foliage (1 to 3 cm). The main soil type is a Cambisol on limestone as bed-rock. The soil pH is weakly acidic (5.1 ± 1.1; mean ± SD).

In October 2019, we collected senescing leaves and needles from 60 tree individual (12 tree species, five true replicates (trees), minimum 200-g leaves or needles per tree individual). These tree species include 8 broadleaved (including *Acer pseudoplatanus*, *Carpinus betulus*, *Fagus sylvatica*, *Fraxinus excelsior*, *Populus* sp., *Prunus avium*, *Quercus robur*, and *Tilia cordata*) and 4 coniferous tree species (including *Picea abies*, *Larix decidua*, *Pinus sylvestris*, and *Pseudotsuga menziesii*). Sampling was carried out with gloves and sterilized plastic bags, and leaves and needles from each tree were separately packed and transported under cooled conditions to the lab. In the laboratory, each leaf and needle were frozen at − 80 °C for subsequent molecular approaches.

### DNA Extraction and Illumina Sequencing

Healthy-looking leaves (up to 10 leaves per tree individual depending on the size of the leaves) and needles (from five branches per tree individual) were subsampled and prepared for DNA extraction. Briefly, we removed loosely adherent dust particles and microbes from leaf and needle samples by vortexing them with a maximum speed for 5 min in sterile Tween solution (0.1% vol/vol), and this step was repeated three times. The samples were then washed three to five times using deionized water. Finally, leaf and needle samples were incubated for 1 h in sterile water at room temperature. Each composite sample was then ground using liquid nitrogen and pestle, homogenized, then stored at − 20 °C for further analysis. Fungal community attached firmly to the leaf and needle samples (~ 120 mg homogenized leaves and needles) was then subjected to DNA extraction using DNeasy PowerSoil Kit (Qiagen, Hilden, Germany) and a Precellys 24 tissue homogenizer (Bertin Instruments, Montigny-le-Bretonneux, France) according to the manufacturer’s instructions. The presence and quantity of genomic DNA were checked using NanoDrop ND-1000 spectrophotometer (Thermo Fisher Scientific, Dreieich, Germany), and the extracts were stored at − 20 °C. Leaf- and needle-associated fungi were characterized by fungal internal transcribed spacer (ITS)–based amplicon sequencing on the Illumina MiSeq sequencing platform, as outlined earlier [30]. For establishing fungal amplicon libraries, the fungal ITS2 gene was amplified using the fungal primer pair fITS7 [5-GTGARTCATCGAATCTTTG-3] [31] and ITS4 primer [5-TCCTCCGCTTATTGATATGC-3] [32] with Illumina adapter sequences. Amplifications were performed using 20-µL reaction volumes with 5 × HOT FIRE Pol Blend Master Mix (Solis BioDyne, Tartu, Estonia). The amplified products were visualized by gel electrophoresis and purified using an Agencourt AMPure XP kit (Beckman Coulter, Krefeld, Germany). Illumina Nextera XT Indices were added to both ends of fungal amplicons. The products from three technical replicates were then pooled in equimolar concentrations. Paired-end sequencing (2 × 300 bp) was performed on the pooled PCR products using a MiSeq Reagent kit v3 on an Illumina MiSeq system (Illumina Inc., San Diego, CA, USA) at the Department of Soil Ecology, Helmholtz Centre for Environmental Research, Germany.

### Bioinformatics

The ITS rDNA sequences corresponding to the forward and reverse primers were trimmed from the demultiplexed raw reads using cutadapt [33]. Paired-end sequences were quality-trimmed, filtered for chimeras, and merged using the DADA2 package [34] through the pipeline dadasnake [30]. Assembled reads fulfilling the following criteria were retained for further analyses: a minimum length of 70 nt, quality scores at least equal to 9 with maximum expected error score of 5 for forward and reverse sequences, and no ambiguous nucleotides. Merging was conducted with 2 mismatches allowed and a minimum overlap of 20 nucleotides required for fungal sequences. High-quality reads were clustered into 2480 amplicon sequence variants (ASVs) for fungi after chimera removal. Fungal ASVs were classified against the UNITE v7.2 database [35]. Set of ASVs were classified using the Bayesian classifier as implemented in the mothur classify.seqs command, with a cut-off of 60. The ASV method is used to infer the biological sequences in the sample, as described previously [36]. Rare ASVs (singletons), which potentially represent artificial sequences, were removed. The dataset was then rarefied. Finally, we obtained 2451 rarefied fungal ASVs with the minimum sequencing depths of 21,967 sequences per sample. Presence/Absence datasets for fungi were used in the statistical analyses. The rarefaction curves of all the samples are provided in the Supplementary Figure S1. The fungal ecological function of each ASV was determined using FUNGuild [21] and FungalTraits [29] according to the authors’ instructions.

### Physiochemical analyses

Wet leaf and needle samples were shaken for 1 h in falcon tubes with 30 mL milliQ water to leach water-soluble components from their surfaces. The leachates were centrifuged for 5 min at 3500 rpm, decanted, and filtered through pre-flushed 0.45-µm regenerated cellulose syringe filters. The remaining leaf/needle material was dried for two weeks at 40 °C for dry weight determination. All quantification results are given in reference to the dry weight. The pH of the leachates was determined using pH paper with a scale precision of 0.2 pH units. N_org_ was calculated as the difference: N_org_ = TN_b_ – N_min_. TN_b_ was analyzed using a sum parameter analyzer with high temperature combustion and chemiluminescence detection (Mitsubishi TN-100; a1 envirosciences, Düsseldorf, Germany). For N_Min_ quantification, a flow injection analyzer (Quikchem QC85S5; Lachat Instruments, Hach Company, Loveland CO, USA) with corresponding manifolds for the nitrogen measurement of ammonium ${\mathrm{N}}_{{\mathrm{NH}}_{4}^{+}}$, nitrite ${\mathrm{N}}_{{\mathrm{NO}}_{2}^{-}}$, and nitrate- plus nitrite ${\mathrm{N}}_{{\mathrm{NO}}_{3}^{-}+{\mathrm{NO}}_{2}^{-}}$ was used. DOC was quantified as non-purgeable organic carbon (NPOC) with a sum parameter analyzer using high-temperature combustion and infrared detection (vario TOC cube, Elementar Analysensysteme GmbH, Langenselbold, Germany). Nutrient ions, Ca, Fe, K, Mg, and P content were determined using Inductively Coupled Plasma–Optical Emission Spectrometry (ICP–OES, PerkinElmer Inc., Waltham, MA, USA) according to manufacturers’ specifications. All method details on physiochemical analyses are provided in supplementary material.

### Statistical analysis

The datasets were tested for normality using the Jarque–Bera JB test and for the equality of group variances using *F*-test (for two datasets) and Levene’s test (for more than two datasets). The statistical differences between proportions of functional assignment by FUNGuild and FungalTraits were performed using *T*-test (for normal distributed data) and Mann–Whitney *U* test (for non-normal distributed data). Effects of tree species and tree types on fungal community composition were visualized and tested with cluster analysis (based on presence-absence data, paired group algorithms, and the Jaccard distance measure) and one-way PERMANOVA (based on presence-absence data and the Jaccard distance measure), over 999 permutations were run. The correlation analyses were performed using Pearson’s *r* (for normal distributed data) and Spearman’ *ρ* (for non-normal distributed data). The statistical differences of ASV richness among different tree species were performed using one-way ANOVA with Tukey’s post hoc test or KW test with Mann–Whitney *U* test. All statistical analyses were performed using PAST version 2.17 [37].

## Results

### Overall Performances of Ecological Function Assignments of FUNGuild and FungalTraits

**Fig. 1:**
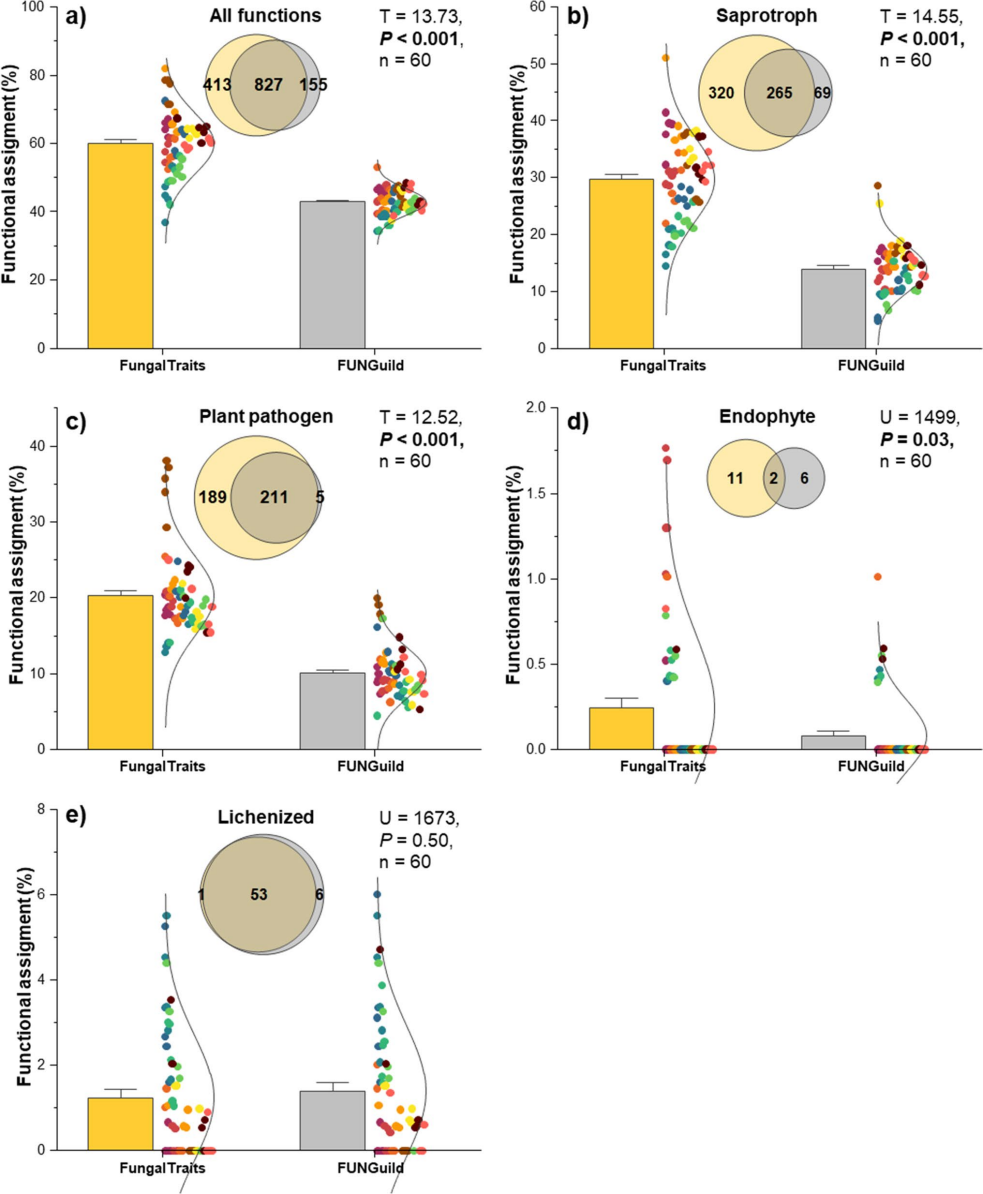
Proportions of functional assignments and Venn diagrams of (**a**) all functions, (**b**) saprotrops, (**c**) plant pathogens, (**d**) endophytes, and (**e**) lichenized fungi by FungalTraits and FUNGuild. The statistical differences were performed using *T*-test (for normal distributed data) and Mann–Whitney *U* test (for non-normal distributed data). Color code of each data point refers to leaves and needles of respective tree species identity and is similar to the color code of Fig. 2. Data used for Venn diagrams are provided in Supplementary Table S3–S6

In total, we assigned functions to 1,395 ASVs (accounted for 57% of total ASVs) using both FUNGuild and FungalTraits annotation tools (Table 1 and Fig. 1). In these total assigned ASVs, 70% and 89% were assigned functions by FUNGuild and FungalTraits, respectively (Table 1 and Fig. 1a). The remaining 30% that were not assigned functions by FUNGuild were assigned solely by FungalTraits and vice versa. 977 ASVs (~ 40% of total ASVs) could not functionally be assigned by both FUNGuild and FungalTraits. As suggested by Nguyen et al. [21], genera with confidence level of “possible” (in total 349 ASVs) were classified as “uncertained” and excluded from the functional analyses in this study (Table 1 and Supplementary Table S1). Overall, we found that the proportion of the total fungal functional assignment by FungalTraits (average of all tree species = 60%) was significantly higher than those by FUNGuild (average of all tree species = 43%) (Fig. 1a). We found a consistent pattern when considering each fungal guild (including saprotroph, plant pathogen, and endophyte) (Fig. 1b–d). The proportions of the functional assignment of these fungal guilds by FungalTraits were also significantly higher than those by FUNGuild, especially for saprotrophs (FungalTraits = 30% and FUNGuild = 14%), plant pathogens (FungalTraits = 20% and FUNGuild = 10%), and endophytes (FungalTraits = 0.24% and FUNGuild = 0.08%). Considering main fungal guilds, FUNGuild assigned only 53 and 51% of plant pathogens and saprotrophs, respectively. Contrarily, FungalTraits assigned up to 99% of these main fungal guilds. Furthermore, 98 and 79% of plant pathogens and saprotrophs, respectively, assigned by FUNGuild were also assigned by FungalTraits. However, for lichenized fungi, we found no significant difference between the proportions of functional assignment by both annotation tools (*P* > 0.05) (Fig. 1e). FUNGuild and FungalTraits shared 88% of total lichenized fungal ASVs. Remarkedly, all ASVs assigned functions such as animal pathogen/animal parasite, ectomycorrhiza, fungal parasite/mycoparasite, and lichen parasite by FUNGuild were subset of those assigned by FungalTraits (Table 1). Epiphyte was, however, an exception. There was no shared epiphytic ASV between both annotation tools.

**Table 1:** Number of ASVs assigned functions by FUNGuild and FungalTraits

Functions	Total number of ASVs assigned to functions by both annotation tools	Total number of ASVs assigned to functions by FUNGuild	Total number of ASVs assigned to functions by FungalTraits	Shared ASVs
All functions	1395	982	1240	827
Animal pathogen/animal parasite	48	21	48	21
Ectomycorrhiza	13	8	13	8
Endophyte	19	8	13	2
Epiphyte	50	5	45	0
Ericoid mycorrhiza	1	1	NA	NA
Fungal parasite/mycoparasite	57	7	57	7
Lichen parasite	18	6	18	6
Lichenized fungi	60	59	54	53
Multifunction	317	317	NA	NA
Plant pathogen	405	216	400	211
Saprotroph	654	334	585	265
Sooty mold	7	NA	7	NA
Unassigned	1354	1120	1211	977
Uncertained (FUNGuild with confidence level of “possible”)	349*	349*	0	0

^*^79 ASVs are shared ASVs between “uncertained” in FUNGuild and “unassigned” in FungalTraits

### Specific Performances of Ecological Function Assignments of FUNGuild and FungalTraits Across 12 Temperate Tree Species

**Fig. 2:**
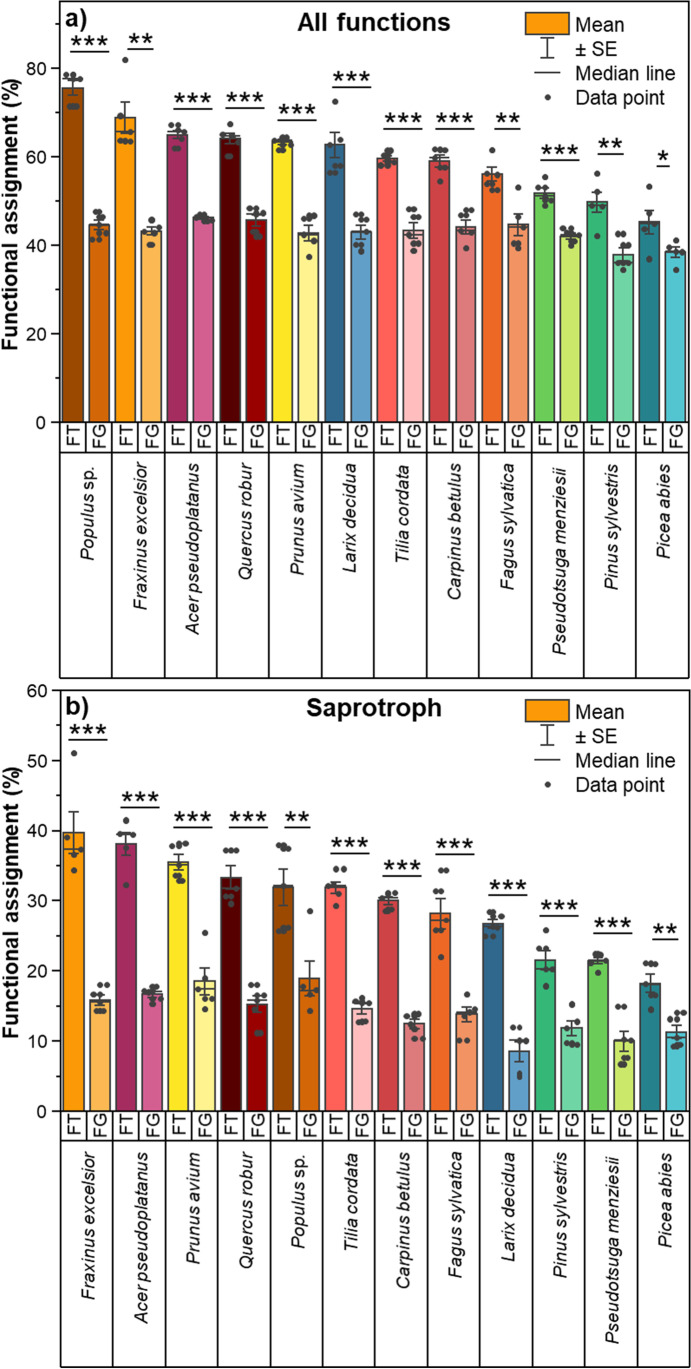
Bar plots of functional assignments of (a) all functions and (b) saprotrophs across 12 tree species. Median of average proportion with standard error are denoted. FT and FG stand for FungalTraits and FUNGuild, respectively. Yellow–red-brown color tone refers to the broadleaved tree species and blue-green color tone the coniferous tree species. The statistical differences between proportions of functional assignments by FungalTraits and FUNGuild in each tree species were performed using *T*-test (*P* < 0.05 = *, *P* < 0.01 = **, *P* < 0.001 = ***)

Based on individual tree species, we also found that FungalTraits provided a significantly higher proportion of the total fungal functional assignment compared to FUNGuild (Fig. 2). This pattern was consistent across all 12 tree species. Among broadleaved tree species, *Populus* sp. revealed the highest proportion of the total fungal functional assignments by FungalTraits, whereas *F. sylvatica* has the lowest proportion (Figs. 1 and 2). Remarkably, the differences between these proportions assigned by both annotation tools were much higher in broadleaved tree species compared to coniferous tree species. The highest difference was found in broadleaved *Populus* sp. (31%), while the lowest difference was observed for *P. abies* (7%). While the proportions of overall fungal functional assignments by FungalTraits were much higher in broadleaved tree species compared to coniferous tree species, FUNGuild provided similar proportions across all tree species. The consistent patterns were found in saprotrophs. The proportions of saprotrophic fungal functional assignments by FungalTraits were significantly higher than those by FUNGuild across 12 tree species. The differences between them were also higher in broadleaved tree species (13–24%) compared to coniferous tree species (7–18%).

### FUNGuild vs. FungalTraits: Interpretation of Richness and Community Composition of Main Fungal Guilds

**Fig. 3:**
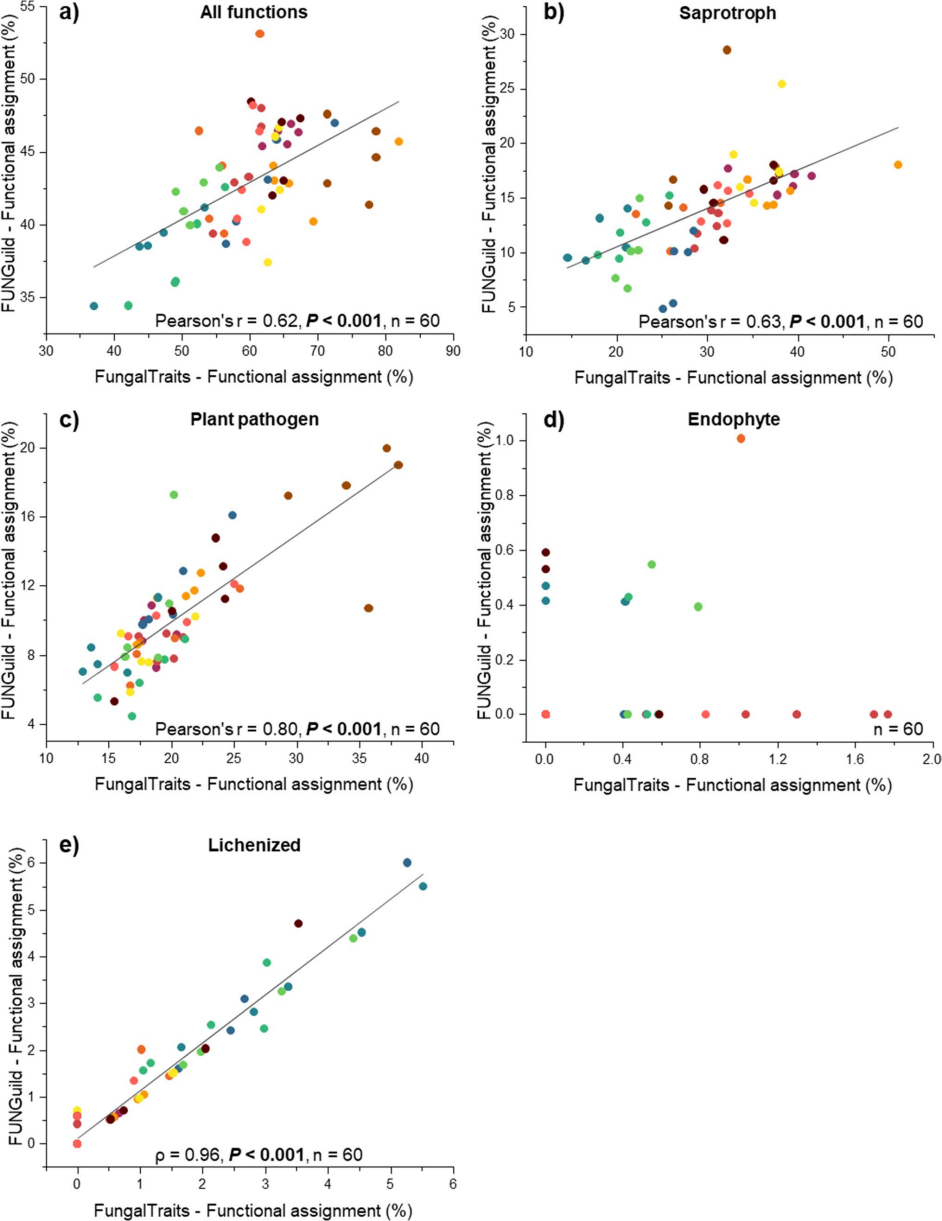
Linear regressions between proportions of functional assignments by FungalTraits and FUNGuild for (**a**) all functions, (**b**) saprotrophs, (**c**) plant pathogens, (**d**) endophytes, and (**e**) lichenized fungi. The statistical differences were performed using Pearson’s *r* (for normal distributed data) and Spearman’ *ρ* (for non-normal distributed data)

We found a significant correlation between the percentage points of total functional assignments by FUNGuild and FungalTraits (Pearson’s *r* = 0.62, *P* < 0.001) (Fig. 3a). However, the correlations were different among fungal guilds. We found that lichenized fungi revealed the highest significant correlation between proportions of functional assignment by FUNGuild and FungalTraits (*ρ* = 0.96, *P* < 0.001), followed by plant pathogens (Pearson’s *r* = 0.80, *P* < 0.001). A significant, but lower correlation, was found for saprotrophs (Pearson’s *r* = 0.63, *P* < 0.001). Contrarily, no correlation was observed for endophytes (*P* > 0.05).

**Fig. 4:**
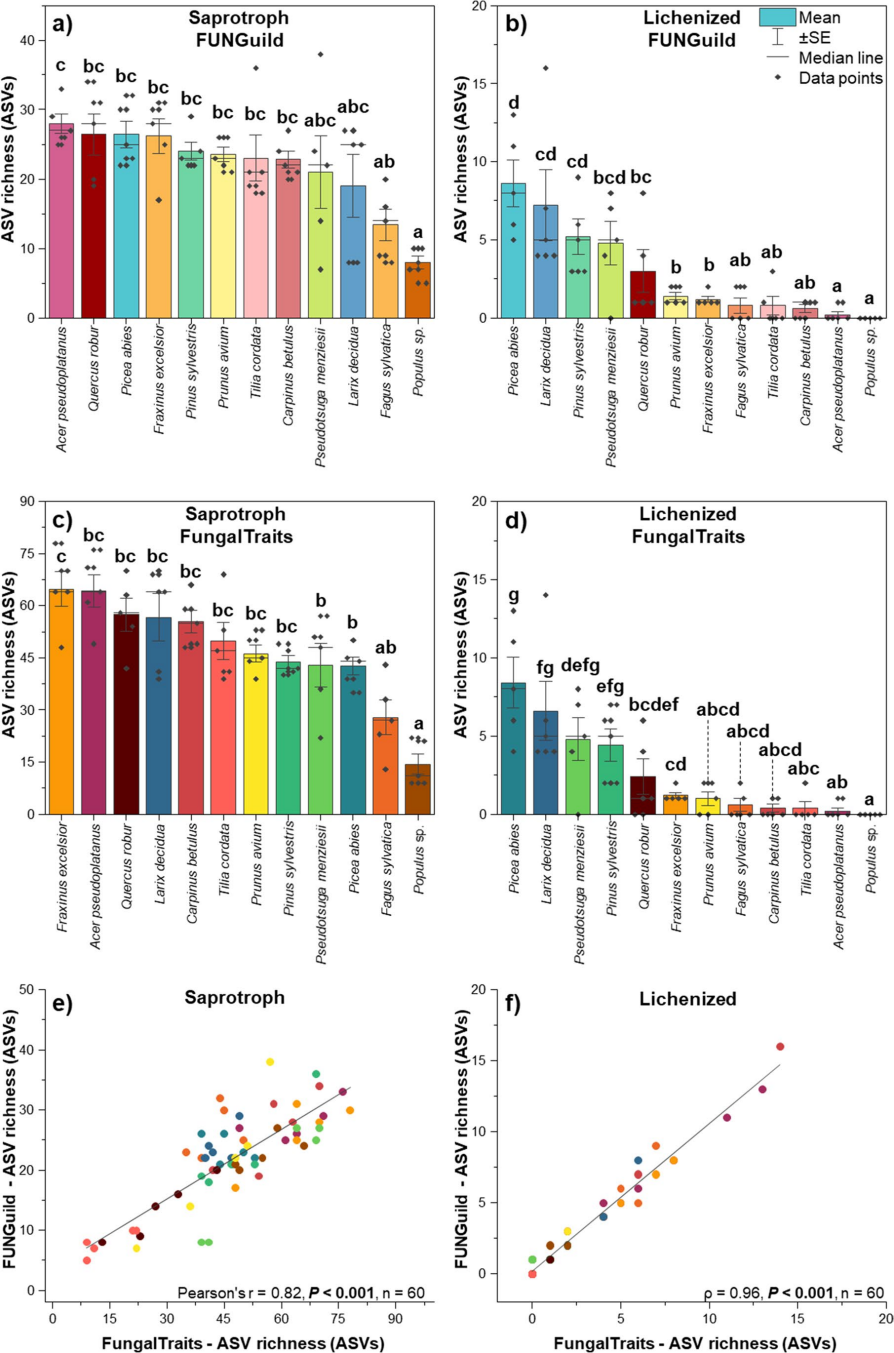
Average ASV richness of fungal saprotrophs (**a**,** c**) and lichenized fungi (**b**, **d**) across 12 tree species obtained by FUNGuild (**a**, **b**) and FungalTraits (**c**, **d**) and correlations between ASV richness values obtained by FUNGuild and FungalTraits for fungal saprotrophs (**e**) and lichenized fungi (**f**). Median of average proportion with standard error are denoted. Yellow–red-brown color tone refers to the broadleaved tree species and blue-green color tone refers to the coniferous tree species. The statistical differences of ASV richness among different tree species were performed using ANOVA or KW test. Correlations were determined using Pearson’s *r* and Spearman’s rank correlation coefficients

**Fig. 5:**
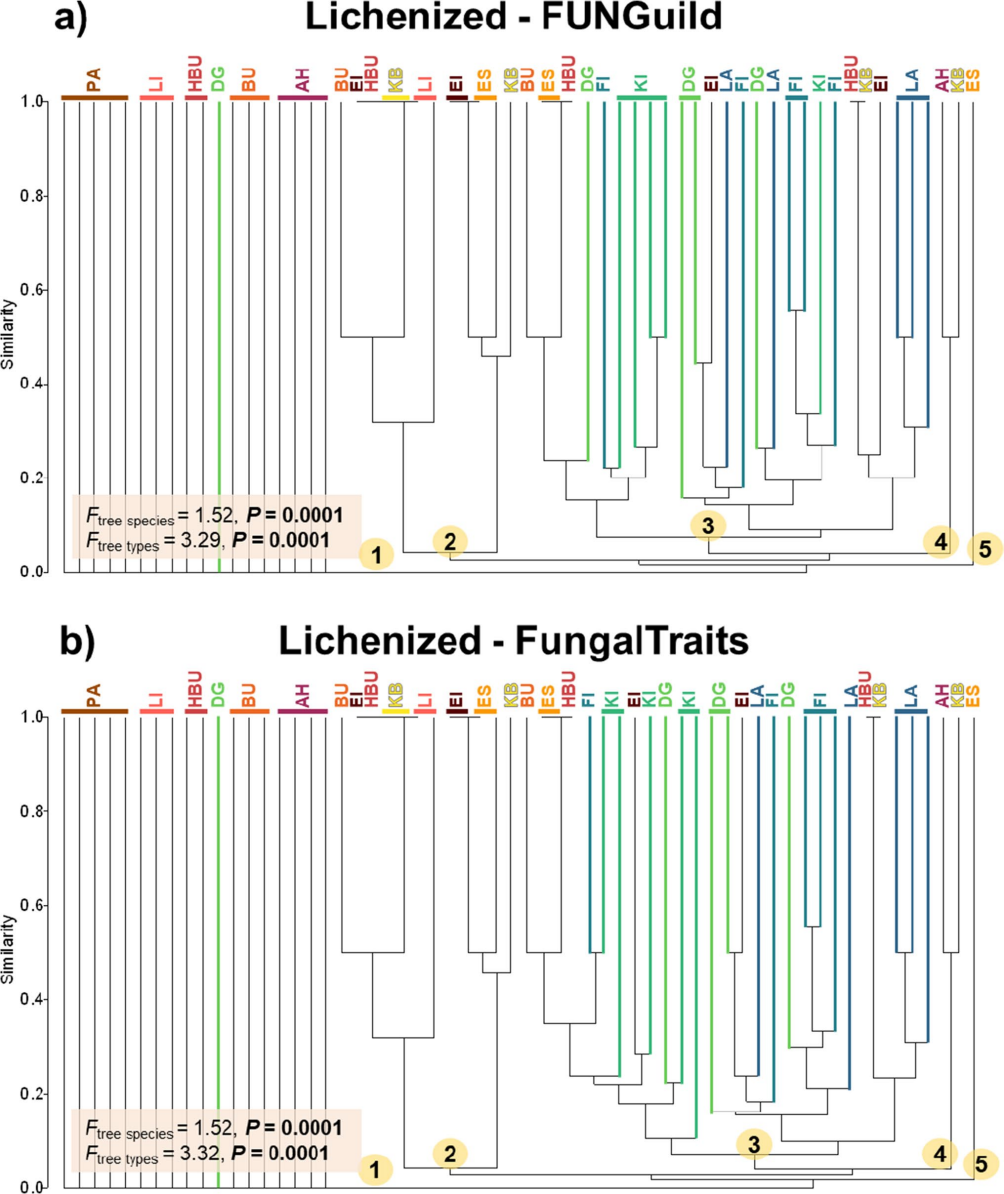
Community composition of lichenized fungi derived from FUNGuild (**a**) and FungalTraits (**b**) of five independent leaf/needle replicates. Yellow–red-brown color tone refers to the broadleaved tree species and blue-green color tone the coniferous tree species. Numbers in yellow circle indicate different clades in the dendrogram for lichen cluster analysis. Species abbreviations for broadleaved tree species are as follows: AH: *Acer pseudoplatanus*, BU: *Fagus sylvatica*, EI: *Quercus robur*, ES: *Fraxinus excelsior*, HBU: *Carpinus betulus*, KB: *Prunus avium*, LI: *Tilia cordata*, and PA: *Populus* sp., and coniferous tree species are: DG: *Pseudotsuga menziesii*, FI: *Picea abies*, KI: *Pinus sylvestris*, and LA: *Larix decidua*. Effects of tree species and tree types were tested with one-way PERMANOVA

**Fig. 6:**
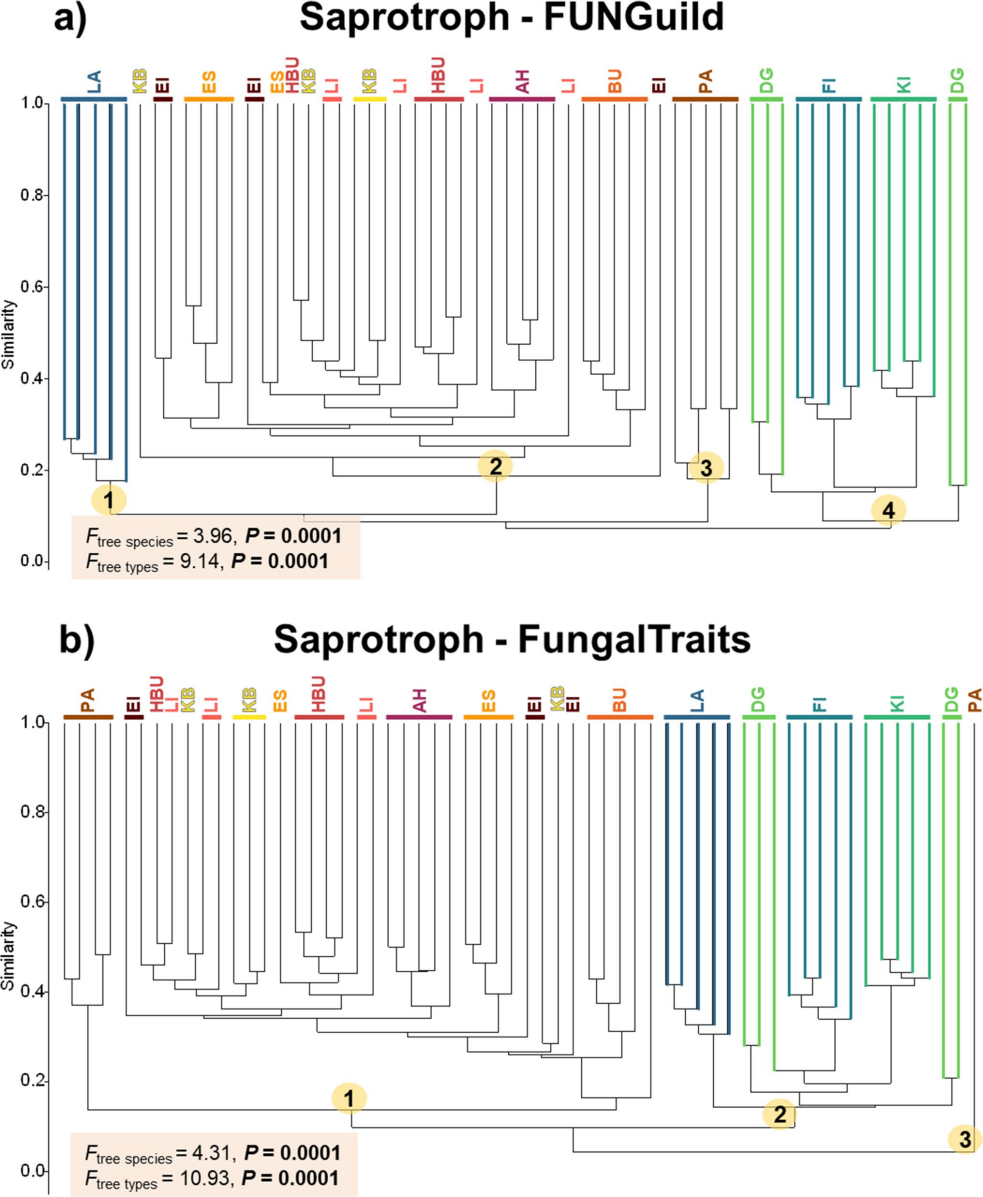
Community composition of saprotrophic fungi derived from FUNGuild (**a**) and FungalTraits (**b**) of five independent leaf/needle replicates. Yellow–red-brown color tone refers to the broadleaved tree species and blue-green color tone the coniferous tree species. Numbers in yellow circle indicate different clades in the dendrogram for saprotroph cluster analysis. Species abbreviations for broadleaved tree species are: AH: *Acer pseudoplatanus*, BU: *Fagus sylvatica*, EI: *Quercus robur*, ES: *Fraxinus excelsior*, HBU: *Carpinus betulus*, KB: *Prunus avium*, LI: *Tilia cordata*, and PA: *Populus* sp., and coniferous tree species are: DG: *Pseudotsuga menziesii*, FI: *Picea abies*, KI: *Pinus sylvestris*, and LA: *Larix decidua*. Effects of tree species and tree types were tested with one-way PERMANOVA

Likewise, the consistent results of the effect of tree species and tree types (broadleaved vs. coniferous trees) on richness and community composition were highly correlated to fungal guilds (Figs. 4, 5, and 6). For lichenized fungi, such results from FUNGuild and FungalTraits are almost identical. We found that needles of all coniferous trees harboured significantly higher richness of lichenized fungi as compared to the broadleaved trees (Fig. 4). An exception was found for *Q. robur*, which exhibited similar richness as compared with *L. decidua*, *P. sylvestris*, and *P. menziesii*. *Picea abies* and *Populus* sp. harboured highest and lowest richness. For fungal community composition, we found that almost all needle samples of coniferous trees clustered together into one clade with few broadleaved trees (Fig. 5). The majority of leaf samples from broadleaved trees (*Populus* sp., *A. pseudoplatanus*, *F. sylvatica*, *T. cordata*) contained no lichenized fungi and clustered together on the left side of the plot. The effects of tree species (*F*_tree species, FG_ = 1.52, *P* = 0.0001 and *F*_tree species, FT_ = 1.52, *P* = 0.0001, respectively) and tree types (*F*_tree type, FG_ = 3.29, *P* = 0.0001 and *F*_tree type, FT_ = 3.32, *P* = 0.0001, respectively) on lichenized fungal community composition based on FUNGuild and FungalTraits were almost identical.

On the other hand, for saprotrophs, the results from FUNGuild and FungalTraits were partly inconsistent (Figs. 4 and 6). Both functional annotation tools showed that tree species and tree types significantly affect the richness of saprotrophic fungi and *Populus* sp. harboured the lowest richness. However, FUNGuild and FungalTraits showed that *A. pseudoplatanus* and *F. excelsior*, respectively, harboured the highest saprotrophic richness (Fig. 4). Nevertheless, we found strong correlations for all tree species of both saprotrophic (Pearson’s *r* = 0.82, *P* < 0.001) and lichenized (*ρ* = 0.96, *P* < 0.001) fungal richness obtained by FUNGuild and FungalTraits (Fig. 4e and f). For saprotrophic fungal community composition derived from FUNGuild and FungalTraits, we found that all leaf samples of coniferous trees separated from broadleaved trees (Fig. 6). However, based on FUNGuild, we detected two clades belong to broadleaved trees (*Populus* sp. separated from other broadleaved trees) and two clades belong to coniferous trees (*L. decidua* separated from other coniferous trees) (Fig. 6a) whereas based on FungalTraits, we detected two clades, each belonged to coniferous and broadleaved trees (one *Populus* sp. sample separated from other trees) (Fig. 6b).

### FUNGuild vs. FungalTraits: Factors Shaping Community Composition of Main Fungal Guilds

We found similar patterns of factors shaping the saprotrophic community composition derived from FUNGuild and FungalTraits. Here, tree species and tree type were the main factors shaping the saprotrophic community composition (Table 2). Besides tree species and tree type, we also found that water content, the majority of water-leachable leaf/needle nutrient compounds (DOC, organic (N_Org_) and inorganic (mineralized) N (N_Min_, ${\mathrm{N}}_{{\mathrm{NH}}_{4}^{+}}$, and ${\mathrm{N}}_{{\mathrm{NO}}_{2}^{-}}$) as well as Ca, Fe, Mg, and P content), and location significantly corresponded with saprotrophic community composition of both annotation tools (Table 2). In the endophytic community, where no correlation between proportions of functional assignment by FUNGuild and FungalTraits was detected, unidentical patterns of factors were found (Table 2). Tree species was the main factor shaping endophytic community composition of both annotation tools along with P content. However, when FUNGuild was employed, we obtained longitude as another main factor significantly corresponded with endophytic community composition. On the other hand, when FungalTraits was applied, Ca and K content were found to be additional factors that significantly corresponded with endophytic community composition (Table 2).

**Table 2:** Goodness-of-fit statistics (*R*^2^) of environmental variables fitted to the nonmetric multidimensional scaling (NMDS) ordination of saprotrophic and endophytic fungal community based on presence/absence data and Jaccard distance measure. Bold letter indicates statistical significances

	Saprotroph				Endophyte			
	FUNGuild		FungalTraits		FUNGuild		FungalTraits	
	*R*^2^	*P*	*R*^2^	*P*	*R*^2^	*P*	*R*^2^	*P*
Tree species	**0.86**	**0.001**	**0.89**	**0.001**	**0.74**	**0.044**	**0.77**	**0.007**
Tree type	**0.51**	**0.001**	**0.61**	**0.001**	0.01	0.886	0.08	0.304
Water content	**0.11**	**0.042**	**0.14**	**0.020**	0.02	0.949	0.15	0.339
pH	0.06	0.184	0.08	0.090	0.41	0.213	0.19	0.199
DOC	**0.50**	**0.001**	**0.54**	**0.001**	0.15	0.613	0.26	0.107
${\mathrm{N}}_{{\mathrm{NH}}_{4}^{+}}$	**0.15**	**0.008**	**0.23**	**0.004**	0.19	0.538	0.20	0.202
${\mathrm{N}}_{{\mathrm{NO}}_{2}^{-}}$	**0.42**	**0.001**	**0.41**	**0.001**	0.05	0.843	0.10	0.469
${\mathrm{N}}_{{\mathrm{NO}}_{3}^{-}}$	0.04	0.311	0.03	0.317	ND	ND	0.25	0.218
N_Min_	**0.16**	**0.008**	**0.20**	**0.006**	0.15	0.607	0.26	0.151
N_Org_	**0.36**	**0.001**	**0.40**	**0.001**	0.03	0.935	0.30	0.085
Ca	**0.50**	**0.001**	**0.53**	**0.001**	0.01	0.956	**0.39**	**0.016**
Fe	**0.15**	**0.009**	**0.20**	**0.001**	0.03	0.929	0.03	0.808
K	0.01	0.695	0.04	0.329	0.59	0.074	**0.44**	**0.017**
Mg	**0.26**	**0.003**	**0.31**	**0.001**	0.12	0.708	0.31	0.059
P	**0.30**	**0.001**	**0.31**	**0.001**	**0.68**	**0.034**	**0.40**	**0.018**
Latitude	**0.65**	**0.001**	**0.66**	**0.001**	0.02	0.938	0.14	0.296
Longitude	**0.17**	**0.007**	**0.15**	**0.010**	**0.87**	**0.004**	0.24	0.124

Variation partitioning analysis revealed similar results for saprotrophs derived from both FUNGuild and FungalTraits (Supplementary Table S2). Tree species alone explained the largest variation (45% of total explainable variance) in the saprotrophic community composition of both annotation tools, followed by leaf/needle nutrients (FUNGuild: 6% and FungalTraits: 3% of total explainable variance). Water content/pH and location alone did not explain the saprotrophic community compositions. The combinations of these factors revealed similar percentage of total explainable variance in saprotrophic community composition of both annotation tools. In the endophytic community composition obtained from FUNGuild, tree species, leaf/needle nutrients, location, and the combination of these three factors explained 46%, 26%, 18%, and 10%, respectively, of total explainable variance (Supplementary Table S2). In the endophytic community composition obtained from FungalTraits, only two factors (tree species and leaf/needle nutrients) were used to explain the variation (Supplementary Table S2). Sixty-six percent, 18%, and 16% of total explainable variance were explained by tree species, leaf/needle nutrient, and the combination of these two factors, respectively.

## Discussion

### Performance of Ecological Function Assignments of FUNGuild and FungalTraits

FUNGuild has been routinely used for functional annotations of mycobiome members across different ecosystems and biomes encompassing both terrestrial and aquatic environments [23, 38–42]. Many studies detected specific responses of a defined set of fungal guilds to different environmental factors which cannot be detected based on the total community analyses [40, 43]. A recent study based on total and active microbiome also illustrates that some ecosystem functions are related to the changes in richness, abundances, and community composition of specific fungal guilds [39]. While FUNGuild is based on a publicly available Python script to annotate fungal functions, FungalTraits works in similar way with the same Python script but offers a more user-friendly Excel-based database and a web-based interface for users without Python expertise [29]. As FungalTraits proof-checked all entries from FUNGuild and included a large set of additional entries, the database FungalTraits encompasses a more comprehensive and faster annotation. FungalTraits receives high attention in the scientific community and has been already applied for investigating fungal guilds in some terrestrial ecosystems [44, 45]. Due to the large number of previous studies using FUNGuild, it is necessary to compare the performance and the ecological interpretation provided by these two annotation tools with high scientific reputation. FungalTraits has been applied together with FUNGuild to annotate functional groups of fungi associated with Orchidaceae [45]. The authors of that study successfully demonstrated symbiont switching and shifts of trophic mode of fungi associated with Orchidaceae. FungalTraits was also successfully applied to annotate the fungal guilds for wood-inhabiting fungi [44]. In this current study, we used the data on mycobiome associated with senescing leaves and needles of 12 temperate tree species to compare the performance and the results obtained by FUNGuild and FungalTraits. Our results clearly show that FungalTraits outperforms FUNGuild in terms of percent functional assignment quantity and quality. The average value of percent functional assignment of FungalTraits reaches 60% and in *Populus* sp., such value reaches 76%. The average value of percent functional assignment of FUNGuild is 43% (ranging from 38–46%). These values are consistent with the percentage points of functional assignment of FUNGuild reported before in many publications [39, 46, 47]. Nevertheless, it is known that the percentage points of functional assignments of taxonomically dependent functional annotation tool highly depends on the quality and quantity of the database backbone of each sequence data, taxonomic identification and functional description [48]. The better performance of FungalTraits is not surprising as it contains a higher number of fungal genera in its database than FUNGuild [21, 29]. Interestingly, we found that the values of percentage points of functional assignments are relatively constant across different tree species when FUNGuild is applied, such value varied greatly when FungalTraits is applied. Furthermore, for FungalTraits-derived datasets, the percentage points of functional assignments are higher for deciduous trees (including broadleaved trees and *L. decidua*) and lower for the remaining coniferous trees (Fig. 2).

### FUNGuild vs. FungalTraits: There Are Some Similarities but also Some Differences

Our current study demonstrates that the quality of functional annotations and the resulting interpretations derived from FUNGuild and FungalTraits are relatively similar; however, they are not identical. Furthermore, the degrees of similarity greatly depend on which fungal guilds were considered. While the results on the effect of tree species and tree types on richness and community composition of lichenized fungi are almost identical when FUNGuild and FungalTraits are applied, for saprotrophic fungi, we could detect some discrepancies between these two annotation tools (Fig. 3). Low discrepancies are expected for plant pathogens as correlations between proportions of percentage points of functional assignments of FUNGuild and FungalTraits are high. In contrast, we expected high discrepancies for endophytes as we detected no correlation (Fig. 3). Such discrepancies derive from the fact that there are mismatches of functional assignments between FUNGuild and FungalTraits. We now identified these mismatches for fungal genera associated with our datasets (Supplementary Table S3–S6). For example, the genus *Fusarium* was assigned to “plant pathogen” as a primary function in FungalTraits. In turn, FUNGuild assigned *Fusarium* to “Animal Pathogen-Endophyte-Lichen parasite-Plant Pathogen-Soil Saprotroph-Wood Saprotroph” with confidence level “possible”. According to the original FUNGuild article, function with confidence level “possible” should be excluded or interpreted with caution. Confidence level “possible” in FUNGuild may contain genera with split ecologies (they perform different or even conflicting functions depending on life stage and environmental conditions). Another example for the mismatch is the identification of genus *Cenangium*. *Cenangium* is classified as “foliar endophyte” in FungalTraits, while it is identified as “saprotroph” with confidence level “probable” in FUNGuild. According to recent research, *Cenangium* was identified as endophyte, saprotroph, and plant pathogen [49, 50], which is consistent with the “Comment on lifestyle” in FungalTraits [29].

Apart from mismatches, FUNGuild identified two or more functions (with confidence level “probable”) for a single fungal taxon; thus, it is difficult to make decision which function or both functions are most likely fitting to this fungus. Nevertheless, it is quite common to report all functions obtained by FUNGuild for a single taxon when the confidence level is at least “probable”. Another possibility for the discrepancy is that FUNGuild can annotate functions to fungi at a higher taxonomic rank (such as family). For example, we found that four fungal ASVs belonged to *Pannariaceae*, *Candelariaceae*, and *Ramalinaceae*, which were annotated as lichenized fungi using FUNGuild (with probable and highly probable confidence levels), but the same ASVs cannot be annotated to any function by FungalTraits. As demonstrated before, FUNGuild and FungalTraits can be applied together to maximize the number of fungal functional annotations and to remove ambiguous annotations. However, based on the results of our datasets, FungalTraits alone already yielded successful functional assignments for high proportions of the fungal community. Adding the functions specifically annotate with FUNGuild to the FungalTraits datasets can increase the percentage points of the total functional assignment by approximately 6%. Apart from the primary lifestyle (function), FungalTraits also provides other interesting information on secondary lifestyle, endophytic interaction capability, plant pathogenic capacity, preferred substrate type, decay type, habitat characteristics, animal biotrophic capacity, hosts, growth form, fruitbody type, Hymenium type, ectomycorrhiza exploration type, ectomycorrhizal lineage, and photobiont.

### FUNGuild vs. FungalTraits: Factors Shaping Community Composition of Main Fungal Guilds

Saprotrophic fungi are among the most important fungal groups driving important ecosystem functions such as accelerating the decomposition rate, enabling the nutrients accessibility and availability for themselves and other microbes [51, 52]. FungalTraits assigns substantially higher number of saprotrophic ASVs (89% of the total assigned saprotrophic ASVs) compared to FUNGuild (51% of the total assigned saprotrophic ASVs). The shared ASVs assigned by both annotation tools account to only ~ 41% of the total assigned saprotrophic ASVs and only moderate correlation is obtained (Pearson’s *r* = 0.63, *P* < 0.001). This is due to the reannotation process of the fungal genera in FungalTraits. Some changes are made to some specific fungal genera by the experts of the field during this process. Nevertheless, we detect similar patterns of factors that shape the saprotrophic community composition derived from both annotation tools. In both datasets, we found that tree species and tree type are the main factors that significantly shape saprotrophic community composition, along with water content and nutrients (water-leachable DOC amount, water-leachable organic and inorganic nitrogen species (${\mathrm{N}}_{{\mathrm{NH}}_{4}^{+}}$, ${\mathrm{N}}_{{\mathrm{NO}}_{2}^{-}}$, N_Min_, N_Org_), Ca, Fe, Mg, and P content), and location. These findings are in line with previously published studies [53, 54]. The microbial macronutrients (such as C, N, and Ca) and transition metal (Fe) have been previously reported to shape the fungal community in different forest management practices [53]. These nutrients are essential elements in macromolecules and also required for many important enzymatic and metabolic processes which are important for microbial growth and activity [53]. In contrast to the results of saprotrophs, the shared ASVs of endophytes account only to 11% of the total assigned endophytic ASVs and no correlation is observed. Longitude is an additional main factor shaping the endophytic community composition of FUNGuild, while Ca and K content solely significantly correspond with those of FungalTraits. The different patterns of significant factors might lead to a different ecological interpretation of endophytes.

### Unexpected High Diversity of Lichens Associated with Senescing Leaves and Needles: Implication and Future Study

In this study, we revealed a high diversity of lichens associated with senescing leaves and needles assigned by both FUNGuild and FungalTraits. In total, we are able to assign 60 lichenized fungal ASVs that were assigned to 21 genera. The proportion of lichenized fungal assignments obtained from both annotation tools is highly correlated and shared ASVs account to ~ 88% of the total assigned lichenized fungal ASVs. FUNGuild provides six more lichenized fungal ASV assignments compared to those of FungalTraits. Among these, four ASVs are not identified at genus level and functions are only assigned on family level. We found a potential controversial issue for the functional assignment of the genus *Sphaerulina* as FUNGuild classifies *Sphaerulina* as lichenized fungi with the confidence level “highly probable”, while FungalTraits assigns in the primary and secondary lifestyles of this genus as “saprotroph” and “plant pathogen”, respectively. A recent study, however, reports *Sphaerulina* as lichenicolous fungi [55], while other studies attributed them as plant pathogen [56, 57]. Therefore, careful double and crosschecking with published datasets is mandatory to verify database outputs especially at low confidence level. FUNGuild provided information on growth morphology and habitat, while FungalTraits additionally provided information on fruitbody type, hymenium type, and primary photobiont of the assigned lichens. Most of the lichens (55 out of 60 ASVs) detected in this study are associated with senescing needles of coniferous tree species (Supplementary Table S1). We observed lichens on the branches of all coniferous tree species used in this study. Our work showed that lichens associated with senescing leaves and needles were both foliicolous lichens (their development starts directly on leaves or needles) and lichens that accidentally grow onto the leaves or needles from the bark of adjacent branches (their development does not start on leaves or needles) [58]. This current work sheds light solely on richness of lichens as influenced by tree species and tree types. Future studies should focus on the effects of tree species, tree types, leaf/needle physicochemical properties, and geographical distances on richness and community composition of lichens.

### FUNGuild vs. FungalTraits: Similarities and Differences of the Assignments of Arbuscular and Ectomycorrhizal Fungi

Our present study emphasizes on the functional assignments and the performances of the annotation tools, FUNGuild and FungalTraits, in the leaf/needle-associated fungal communities. However, in nature, mycorrhizal fungi are also considered as important functional groups associated with plants and play crucial role in promoting their performances [59, 60]. Thus, for the sake of completeness, we further evaluate the functional assignment of arbuscular (AMF) and ectomycorrhizal (EcM) fungi using FUNGuild [21] and FungalTraits [29]. As AMF are mostly derived from the phylum Glomeromycota, we simply compare all 51 AMF genera from both annotation tools. All assigned AMF genera are covered by FungalTraits alone and 20 AMF genera from FungalTraits are absent from the FUNGuild database (Supplementary Table S7). These new 20 AMF genera are erected in previous studies published between the year 2018 and 2019 [61–64]. However, FUNGuild assigns AMF already at family, order, and phylum level (Glomeromycota). For the analysis of EcM, we employ a dataset from a recently published study investigating EcM in soils at different elevation levels (830 and 1300 m a.s.l) [65]. We assign 26 EcM genera by both annotation tools. FungalTraits covers all 26 EcM genera and FUNGuild assigns 25 EcM genera (Supplementary Figure S2). The fungal genus, *Pustularia*, cannot be assigned to the ecological function by FUNGuild. Members of this genus were previously reported as EcM [66, 67]. Nevertheless, both annotation tools refer to the same ecological interpretation (Supplementary Figure S2). The richness and community composition of EcM significantly differ at different elevation levels. On the basis of this information, we conclude that the performances of FUNGuild and FungalTraits in assigning AMF and EcM are not different.

## Conclusions

Functional assignment of fungal amplicon sequencing datasets is of pivotal interest to infer a more mechanistic understanding of phylogenetic information and to ease the assessment of ecosystem processes of the respective habitat. The quantity and quality of fungal functional annotations were significantly better in FungalTraits than in FunGuild for evaluating the functional guilds on senescing leaves and needles of 12 temperate tree species. The transferability to other environments and research tasks should be addressed in upcoming studies.

## Supplementary Information

Below is the link to the electronic supplementary material.

Supplementary file1 (PDF 468 KB)

Supplementary file2 (XLSX 751 KB)

## Data Availability

The ITS rRNA gene sequences were deposited in the National Center for Biotechnology Information (NCBI) Sequence Read Archive under the accession number PRJNA753096.
