# Supplementary material for: FungalTraits vs. FUNGuild: Comparison of Ecological Functional Assignments of Leaf- and Needle-Associated Fungi Across 12 Temperate Tree Species
Source: Microb Ecol. 2022 Feb 5;85(2):411–28. doi: 10.1007/s00248-022-01973-2 (PMC9958157; doi:10.1007/s00248-022-01973-2)
Supplement: Supplementary file 1 — Supplementary file1 (PDF 468 KB) [file 248_2022_1973_MOESM1_ESM.pdf]

Supplementary Material

FungalTraits *vs*. FUNGuild: Comparison of ecological functional assignments of leaf- and needle-associated fungi across 12 temperate tree species.

Benjawan Tanunchai[1,2¶], Li Ji[1,3¶], Simon Andreas Schroeter[4], Sara Fareed Mohamed Wahdan[1,5], Shakhawat Hossen[1,6], Yoseph Delelegn[1], François Buscot[1,7], Ann-Sophie Lehnert[4], Eliane Gomes Alves[4], Ines Hilke[4], Gerd Gleixner[4], Ernst-Detlef Schulze[4], Matthias Noll[2,6*], Witoon Purahong[1*]

[1]UFZ-Helmholtz Centre for Environmental Research, Department of Soil Ecology, Theodor-Lieser-Str. 4, 06120 Halle (Saale), Germany.

[2]Bayreuth Center of Ecology and Environmental Research (BayCEER), University of Bayreuth, Bayreuth, Germany.

[3]Key Laboratory of Sustainable Forest Ecosystem Management-Ministry of Education, School of Forestry, Northeast Forestry University, 150040 Harbin, P.R. China.

[4]Max Planck Institute for Biogeochemistry, Biogeochemical Processes Department, Hans-Knöll-Str. 10, 07745 Jena, Germany.

[5]Botany Department, Faculty of Science, Suez Canal University, 41522 Ismailia, Egypt.

[6]Institute of Bioanalysis, Coburg University of Applied Sciences and Arts, Coburg, Germany.

[7]German Centre for Integrative Biodiversity Research (iDiv) Halle-Jena-Leipzig, Deutscher Platz 5e, 04103 Leipzig, Germany.

*Correspondence: witoon.purahong@ufz.de; Tel.: 0345-5585207; Matthias.noll@hs-coburg.de

¶This author contributed equally.

Physiochemical analyses

Wet leaf and needle samples were shaken for 1 h in falcon tubes with 30 mL milliQ water to leach water-soluble components from their surfaces. The leachates were centrifuged for 5 min at 3500 rpm, decanted and filtered through pre-flushed 0.45 µm regenerated cellulose syringe filters. The remaining leaf/needle material was dried for two weeks at 40 °C for dry weight determination. All quantification results are given in reference to the dry weight. The pH of the leachates was determined using pH paper with a scale precision of 0.2 pH units.

Simultaneous analysis of total bound nitrogen (TN_b) and inorganic nitrogen (N_{min}) was performed to quantify organic nitrogen (N_{org}). N_{org} was calculated as the difference: $N_{org} = TN_b – N_{min}$. TN_b was analyzed using a sum parameter analyzer with high temperature combustion and chemiluminescence detection (Mitsubishi TN-100; a1 envirosciences, Düsseldorf, Germany). An optimal volume of 4 ml per sample was required for all rinsing and analysis procedures. All samples were measured as triplicates. A sample volume of 30 µL each was automatically injected into the combustion tube filled with a platinum catalyst. The samples were combusted at 800°C in pure oxygen. During this process, all organic and inorganic nitrogen compounds in the sample are oxidized to NO_x and subsequently converted to NO. The NO reacts with ozone in the chemiluminescence detector according to the following oxidation reaction: $NO + O_3 \rightarrow NO_2 + O_2 + hv$. The energetically activated NO_2 emits light spontaneously, and the intensity of the emitted light is proportional to the NO concentration. Detection is performed by a photomultiplier. For N_{Min} quantification, a flow injection analyzer (Quikchem QC85S5; Lachat Instruments, Hach Company, Loveland CO, USA) with corresponding manifolds for the measurement of ammonium nitrogen $N_{NH_4^+}$, nitrite nitrogen $N_{NO_2^-}$, and nitrate- plus nitrite nitrogen $N_{NO_3^-+NO_2^-}$ was used. In this instrument, samples and the required chemical reagents are pumped through a system of calibrated tubing and mixed together in a matrix-related carrier stream. After reaction with the reagents, the analytes are photometrically quantified by measuring the absorbance at a special wave length in a flow-through cuvette. An optimal volume of 10 ml per sample was required for all rinsing, dilution and analysis procedures. For this reason, only one single measurement could be performed per sample. $N_{NH_4^+}$ was determined by the gas diffusion method. For this purpose, samples were automatically mixed with an alkaline buffer at the manifold. The resulting NH_3 was passed through a gas diffusion membrane and absorbed into an acidic indicator stream. The resulting pH change causes a color change in the indicator solution, which was measured photometrically at $\lambda = 590$ nm. The intensity of the coloration is proportional to the $N_{NH_4^+}$ concentration in the sample. $N_{NO_3^-}$ was reduced to

nitrite using a cadmium column in the manifold prior to the chemical reaction to form an azo dye. The nitrate reduced by cadmium and the nitrite originally present in the sample were analyzed using the Griess reaction by diazotization with sulfanilamide and coupling with N-(1-naphthyl) ethylenediamine dihydrochloride. The deep pink color of the resulting dye was measured at λ = 520 nm. $N_{NO_2^-}$ alone was determined after the same reaction, without using a cadmium column.

DOC was quantified as non-purgeable organic carbon (NPOC) with a sum parameter analyzer using high-temperature combustion and infrared detection (vario TOC cube, Elementar Analysensysteme GmbH, Langenselbold, Germany). 10 mL of the sample material was transferred to a thermally cleaned glass vial. To remove the inorganic carbon, all samples were acidified directly in the sample vial. Addition of 200 µL of 10% HCl was to ensure that each sample had a pH<2. Prior to analysis, samples were stripped on the autosampler with the carrier gas. The inorganic carbon volatilizes and all non-volatile organic carbon compounds remain in the sample vial. Each sample was measured as triplicate. A sample volume of 200 µL each was automatically injected into the ash finger of the combustion tube which contains platinum as catalyst. The samples were combusted at 850°C in synthetic air, a hydrocarbon-free mixture of nitrogen and oxygen. After removing moisture from the combustion gas, NPOC was quantified by IR detection of CO_2 formed from the organic carbon compounds in the sample. Nutrient ions, Ca, Fe, K, Mg, and P content were determined using Inductively Coupled Plasma–Optical Emission Spectrometry (ICP–OES, PerkinElmer Inc., Waltham, MA, USA) according to manufacturers’ specifications.

Figure S1 Rarefaction curves

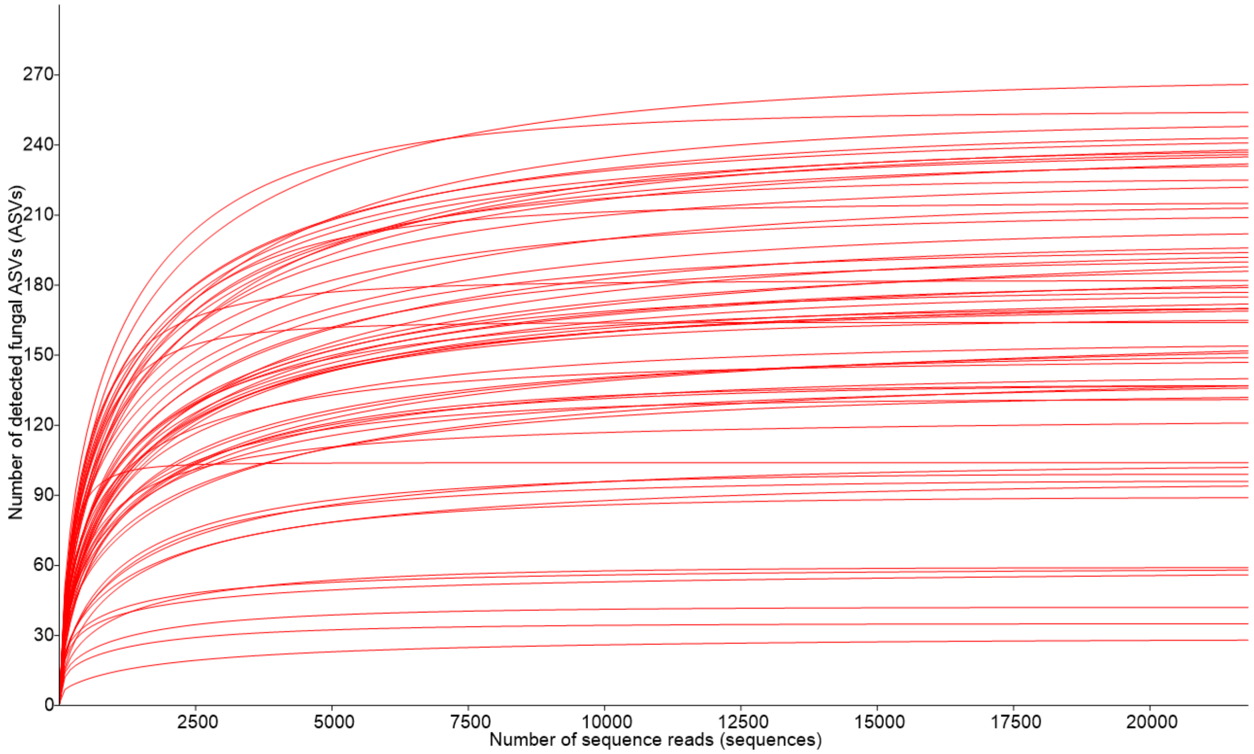

Figure S2 Ectomycorrhiza (EcM) detected in soils of different elevation levels (830 and 1300 m a.s.l.) assigned by FUNGuild and FungalTraits: a) EcM ASV richness ± SE, b) EcM genera assigned by FUNGuild and FungalTraits, and cluster analysis of EcM community composition from c) FUNGuild and d) FungalTraits. This dataset is previously published elsewhere [1]. The statistical differences were accessed using *t*-test: ns = not significant, * = *P* < 0.05.

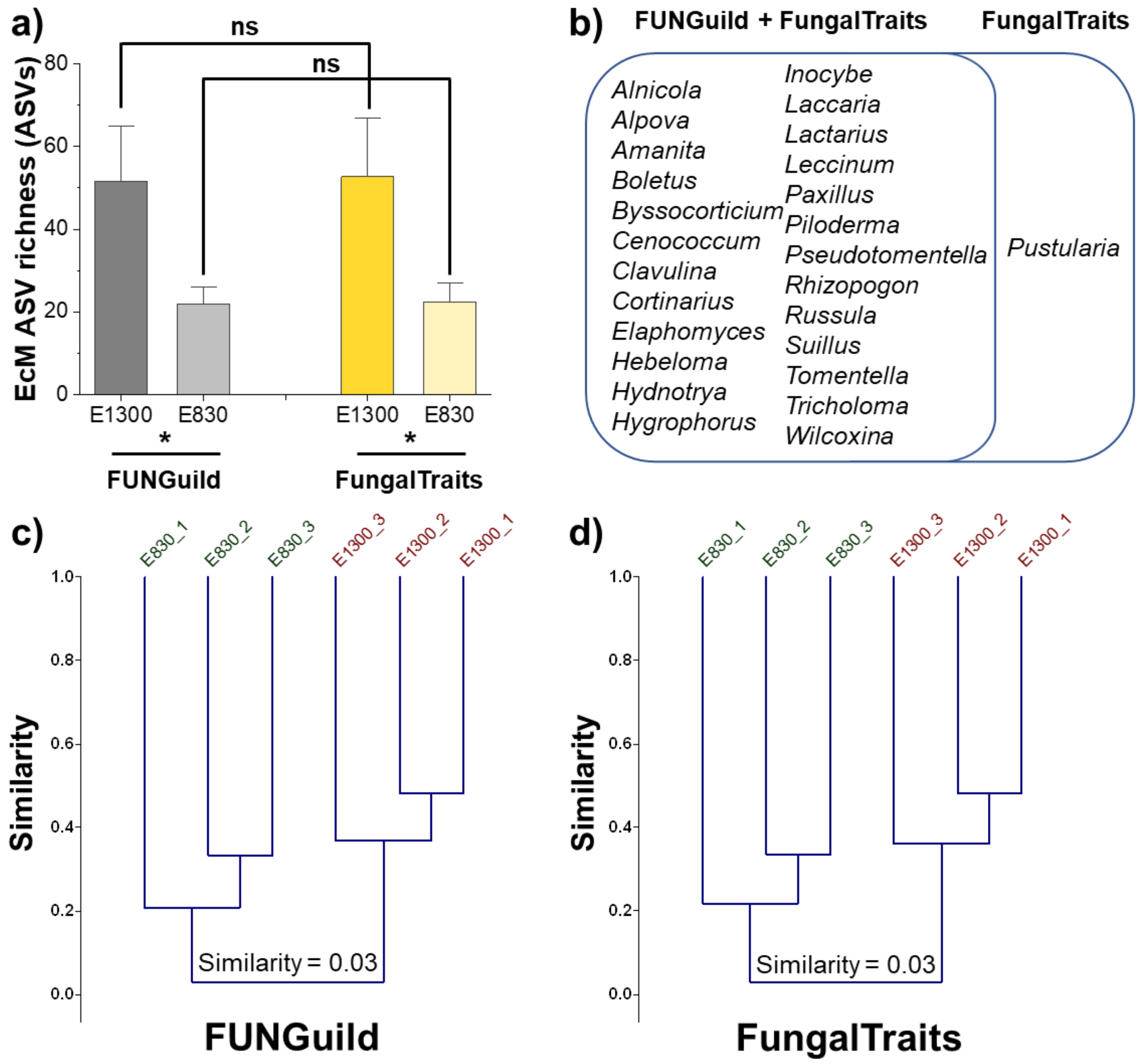

Table S1 Relative abundances (%) of fungal ASVs associated with leaves and needles of 12 temperate forest assigned functions by FUNGuild and FungalTraits (please see in another excel file).

Table S2. Factors explain variations in saprotrophic and endophytic fungal community composition associated with leaves and needles of temperate tree species based on presence/absence data and Jaccard distance measure. The factors used in the variation analysis are the factors that significantly corresponded with the saprotrophic and endophytic fungal community (please see Table 2). ND = Not detected.

Variables	Saprotroph		Endophyte	
	FUNGuild	FungalTraits	FUNGuild	FungalTraits
	Total explainable variance (33%)	Total explainable variance (33%)	Total explainable variance (78%)	Total explainable variance (77%)
Tree species	45	45	46	66
pH / water content	0	0	ND	ND
Leaf/needle nutrients	6	3	26	18
Location	0	0	18	ND
Tree species x pH / water content	0	0	ND	ND
Tree species x Leaf/needle nutrients	15	18	0	16
Tree species x Location	6	6	0	ND
pH / water content x Leaf/needle nutrients	0	0	ND	ND
pH / water content x Location	0	0	ND	ND
Leaf/needle nutrients x Location	0	0	0	ND
Tree species x pH / water content x Leaf/needle nutrients	6	6	ND	ND
Tree species x pH / water content x Location	0	0	ND	ND
Tree species x Leaf/needle nutrients x Location	18	21	10	ND
pH / water content x Leaf/needle nutrients x Location	0	0	ND	ND
Tree species x pH / water content x Leaf/needle nutrients x Location	3	0	ND	ND

Table S3 Saprotrophs assigned by FUNGuild and FungalTraits (please see in another excel file).

Table S4 Plant pathogens assigned by FUNGuild and FungalTraits (please see in another excel file).

Table S5 Endophyte assigned by FUNGuild and FungalTraits (please see in another excel file).

Table S6 Lichenized fungi assigned by FUNGuild and FungalTraits (please see in another excel file).

Table S7 Arbuscular mycorrhizal fungi assigned by FUNGuild and FungalTraits (please see in another excel file).

References

1. Ji L, Shen F, Liu Y, et al (2022) Contrasting altitudinal patterns and co-occurrence networks of soil bacterial and fungal communities along soil depths in the cold-temperate montane forests of China. CATENA 209:105844. https://doi.org/10.1016/j.catena.2021.105844
